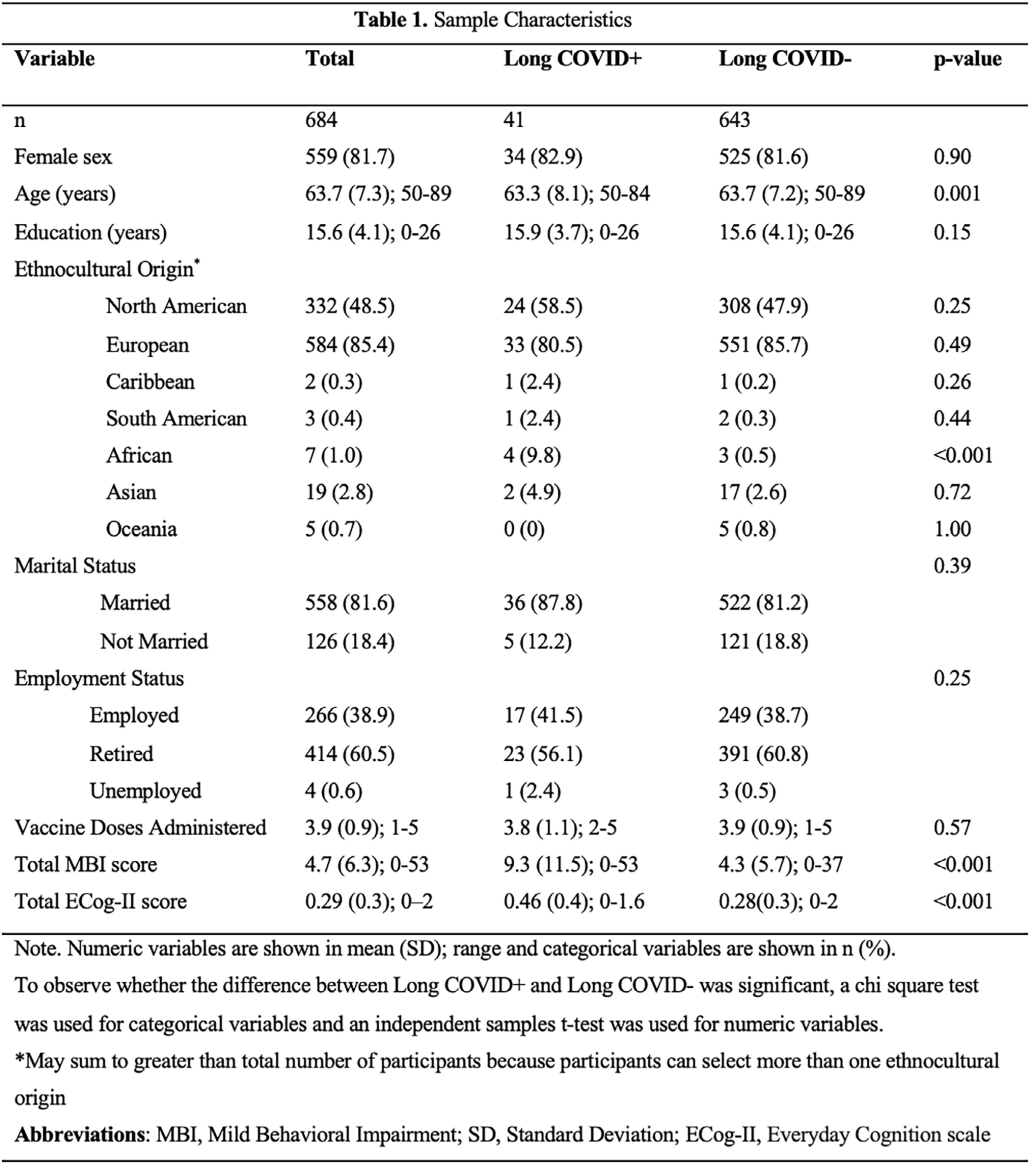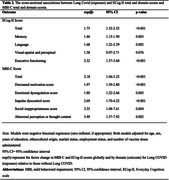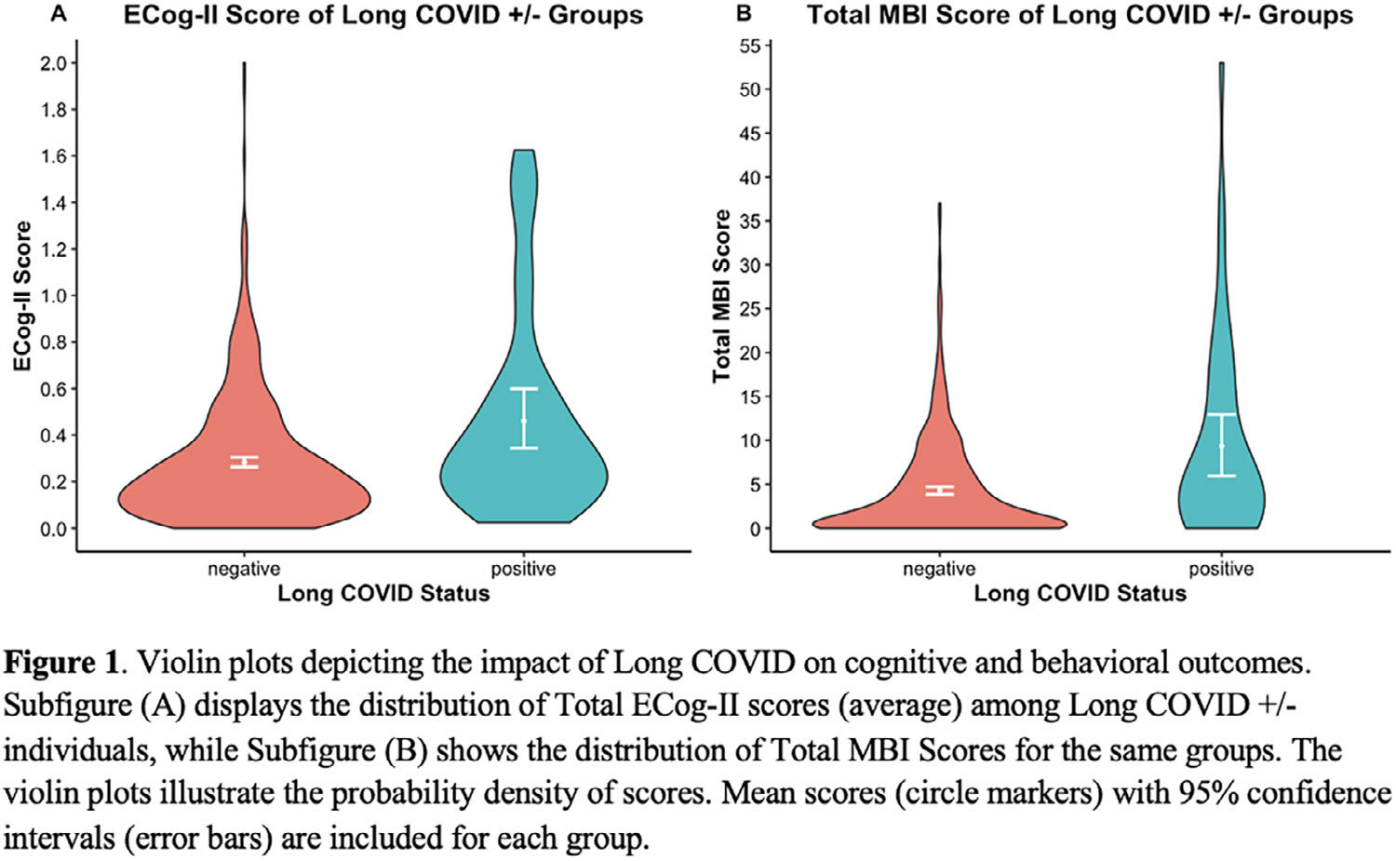# Later‐life cognitive and behavioral changes in older adults with Long COVID

**DOI:** 10.1002/alz.088035

**Published:** 2025-01-09

**Authors:** Sabika Azhar, Dylan X. Guan, Eric E. Smith, Zahinoor Ismail

**Affiliations:** ^1^ University of Calgary, Calgary, AB Canada; ^2^ Hotchkiss Brain Institute, University of Calgary, Calgary, AB Canada; ^3^ Department of Clinical Neurosciences and Hotchkiss Brain Institute, University of Calgary, Calgary, AB Canada

## Abstract

**Background:**

Individuals diagnosed with COVID‐19 may continue to experience symptoms long after infection. Research suggests that the COVID‐19 virus may be linked to brain pathology and dementia risk, possibly due to neurological complications and long‐term cognitive effects. Mild Behavioral Impairment (MBI) is an early indicator of dementia risk characterized by later life onset of persistent changes in behavior or personality. To better understand the consequences of COVID‐19 on older adults, we investigated whether older adults who experienced Long COVID reported more severe later‐life emergent cognitive and behavioral symptoms linked to elevated dementia risk.

**Method:**

Participants were from the Canadian Platform for Research Online to Investigate Health, Quality of Life, Cognition, Behavior, Function, and Caregiving in Aging (CAN‐PROTECT), who self‐reported testing positive for COVID‐19 (n = 684), stratified by whether they reported having Long COVID. The severity of subjective cognitive symptoms was measured with the Everyday Cognition scale (ECog‐II). The severity of MBI symptoms was measured with the MBI Checklist (MBI‐C). We modelled associations between Long COVID (exposure) and ECog‐II or MBI‐C total scores using negative binomial regression models (zero‐inflated, if appropriate), adjusting for age, sex, education, ethnocultural origin, marital status, employment status, and number of vaccine doses administered.

**Result:**

Participants (81.7% female, mean age 63.7±7.3 years) who experienced Long COVID (n = 41) reported an ECog‐II total score 1.73 times higher (95% CI: 1.32‐2.32, p ≤0.001), and an MBI‐C total score 2.18 times higher (95% CI: 1.46‐3.22, p≤0.001) than those without Long COVID. Effect sizes differed for each of the ECog‐II and MBI‐C domains (Table 2). The greatest magnitude of effect was seen in the executive function domain for ECog‐II with a 2.32 times greater score (95% CI: 1.57‐3.60, p≤0.001), and psychotic symptoms domain for MBI with a 3.49 times higher score (95% CI: 1.57‐7.92, p = 0.002), compared to those without Long COVID

**Conclusion:**

Among older adults who tested positive for COVID‐19, those who experienced Long COVID symptoms reported more severe cognitive and behavioral symptoms that indicate elevated risk for dementia. Future studies should investigate symptoms over time and mechanisms linking Long COVID with potential prodromal cognitive and behavioral symptoms for dementia.